# xCT, not just an amino-acid transporter: a multi-functional regulator of microbial infection and associated diseases

**DOI:** 10.3389/fmicb.2015.00120

**Published:** 2015-02-19

**Authors:** Lu Dai, Mairi C. Noverr, Chris Parsons, Johnan A. R. Kaleeba, Zhiqiang Qin

**Affiliations:** ^1^Research Center for Translational Medicine and Key Laboratory of Arrhythmias of the Ministry of Education of China, East Hospital, Tongji University School of Medicine, Shanghai, China; ^2^Department of Medicine, Louisiana State University Health Sciences Center, Louisiana Cancer Research Center, New Orleans, LA, USA; ^3^Department of Oral and Craniofacial Biology, Louisiana State University Health Sciences Center, Louisiana Cancer Research Center, New Orleans, LA, USA; ^4^Department of Microbiology and Immunology, Uniformed Services University of the Health Sciences, Bethesda, MD, USA; ^5^Department of Microbiology, Immunology, and Parasitology, Louisiana State University Health Sciences Center, Louisiana Cancer Research Center, New Orleans, LA, USA

**Keywords:** KSHV, virus, bacteria, xCT, cysteine, glutamate

## Abstract

Expression of xCT, a component of the x_c_^–^ amino-acid transporter, is essential for the uptake of cystine required for intracellular glutathione (GSH) synthesis and maintenance of the intracellular redox balance. Therefore, xCT plays an important role not only in the survival of somatic and immune cells, but also in other aspects of tumorigenesis, including the growth and malignant progression of cancer cells, resistance to anticancer drugs, and protection of normal cells against oxidative damage induced by carcinogens. xCT also functions as a factor required for infection by Kaposi’s sarcoma-associated herpesvirus (KSHV), the causative agent of Kaposi’s sarcoma (KS) and other lymphoproliferative diseases associated with HIV/AIDS. In spite of these advances, our understanding of the role of xCT in the pathogenesis of infectious diseases is still limited. Therefore, this review will summarize recent findings about the functions of xCT in diseases associated with microbial (bacterial or viral) infections, in particular KSHV-associated malignancies. We will also discuss the remaining questions, future directions, as well as evidence that supports the potential benefits of exploring system x_c_^–^ as a target for prevention and clinical management of microbial diseases and cancer.

## BACKGROUND

The x_c_^–^ amino-acid transporter, consisting of xCT (also named as SLC7A11) and its chaperone CD98, functions as a Na^+^-independent electroneutral exchange system for cystine/glutamate (Bannai and Kitamura, 1980). Expression of xCT on the cell membrane is essential for the uptake of cystine required for synthesis of intracellular glutathione (GSH), an anti-oxidant that plays an important role in maintaining the intracellular redox balance (Bannai, 1986; Patel et al., 2004). For most cancer cells, the uptake of cystine/cysteine from the microenvironment is crucial for growth and viability (Lo et al., 2008). Therefore, xCT is highly expressed by a variety of malignant tumors such as lymphoma, glioma, breast carcinoma, and prostate cancer (Gout et al., 2001; Narang et al., 2003; Chung et al., 2005; Doxsee et al., 2007). xCT is also involved in other important cellular functions within cancer cells, such as chemoresistance (Okuno et al., 2003; Huang et al., 2005) and autophagy (Guo et al., 2011). Interestingly, xCT-deficient mice display impaired survival of activated macrophages at the inflammatory site, which subsequently enhances chemically induced tumorigenesis (Nabeyama et al., 2010). In addition, xCT has been associated with some central nervous system (CNS) and eye diseases, such as cerebral ischemia (stroke), trauma, Alzheimer’s disease and retinopathy (Choi, 1987; Bridges et al., 2004; Nagasawa et al., 2005; Qin et al., 2006). Interestingly, we and others recently reported the functional contributions of xCT to the pathogenesis of Kaposi’s sarcoma-associated herpesvirus (KSHV; Kaleeba and Berger, 2006a,b; Qin et al., 2010b, 2013; Dai et al., 2014a), a principal causative agent of several cancers arising in patients with compromised immune systems (Chang et al., 1994). In light of the emerging pleiotropic functions of xCT, the goal of this review is to summarize recent findings about the role of xCT in microbial infection and associated diseases (particularly virus-associated malignancies), and to discuss potential future directions for research in this particular field.

## xCT AND KSHV PATHOGENESIS

### xCT AS A CELLULAR RECEPTOR FOR KSHV ENTRY

Like other well-studied human herpesviruses, KSHV is presumed to enter cells by direct fusion with the target cell membrane as the predominant mode of infection (Dezube et al., 2002; Pertel, 2002; Kaleeba and Berger, 2006a; Longnecker and Neipel, 2007), although endocytosis and other receptor-independent mechanisms of entry have also been described (Akula et al., 2003; Rappocciolo et al., 2008; Greene and Gao, 2009; Garrigues et al., 2014). Using a functional complementary DNA selection assay, we recently identified xCT as an important component of a multi-molecular complex that mediates KSHV entry by direct fusion with the target cell membrane (Kaleeba and Berger, 2006b). The KSHV receptor complex consists of xCT, CD98, and other “outside-in” signaling molecules such as integrins, suggesting that the virus also exploits pre-existing signaling pathways to initiate events that promote virion entry into a variety of cell types. Depending on the cell type, the initial stages of KSHV infection involve target cell recognition through temporal interactions with attachment factors (heparan sulfate and DC-SIGN) or signaling molecules such as integrins (αvβ3, α3β1, and αvβ5); in some cases, these initial steps may be sufficient to mediate virion uptake, or they may be followed by recruitment of the CD98/xCT complex that then mediates viral glycoprotein-mediated fusion of the viral and target cell membranes (Veettil et al., 2008, 2014; Hahn et al., 2009). While these discoveries have advanced our understanding of the early events in the infectious process of KSHV, several questions remain. For example, although recent studies have demonstrated that KSHV can infect some B cell subsets or cell-lines (Rappocciolo et al., 2008; Hassman et al., 2011; Myoung and Ganem, 2011; Dollery et al., 2014), we found that most monocytic and other immortalized lymphoblastoid cell lines did not express detectable levels of xCT mRNA and are generally refractory to KSHV infection. We also failed to detect xCT mRNA in human CD19^+^ primary B cells isolated from fresh peripheral blood mononuclear cells (PBMCs), which is paradoxical given that the virus is etiologically linked to two B cell lymphoproliferative disorders, namely multicentric Castleman’s disease (MCD) and primary effusion lymphoma (PEL; Cesarman et al., 1995; Soulier et al., 1995). Remarkably, ectopic expression of recombinant xCT does not cure the poor target susceptibility of B cell lines to KSHV, whereas over-expression of xCT in K562 cells and other cells that express α3, β1, αv, or β3 integrin subunits can render these cells more susceptible to KSHV, suggesting that the receptor function of xCT is more demonstrably robust in adherent and other cells that also express the relevant integrin co-receptor complexes that provide the requisite supporting role for efficient virion adsorption and signaling prior to virion entry. Interestingly, a recent study found that activated blood and tonsillar B cells can be productively infected with KSHV, which can be blocked by the pretreatment of the cells with antibody specific for DC-SIGN or with the selective DC-SIGN inhibitor, mannan, but not antibody specific for xCT (Rappocciolo et al., 2008). We also recently demonstrated that heparan sulfate (HS), not xCT, may be required for KSHV infection of human primary oral fibroblasts (Dai et al., 2014b). Together, these findings suggest that the receptor function of xCT may be cell-context dependent and is presumably determined by physiologic triggers of receptor expression within the target site, as well as the relative surface expression of various cell-type specific cofactors and other accessory molecules that must be recruited to the virion attachment site.

### xCT AND SURVIVAL OF KSHV-INFECTED CELLS

The biological relevance of xCT in KSHV pathogenesis goes beyond its function as a virus receptor. As mentioned above, xCT naturally functions as a transporter for extracellular cystine that is then used in the synthesis of GSH, an anti-oxidant that restores the intracellular redox balance and protects cells from death induced by reactive oxygen species (ROS) or reactive nitrogen species (RNS; Bannai, 1986). As an adaptive response, xCT is upregulated when intracellular levels of GSH are low, such as in settings of oxidative stress among late stage HIV-AIDS patients who are also likely to experience more aggressive KS (Mallery et al., 2004; Ma et al., 2009, 2013). Accordingly, we found that xCT is upregulated within more advanced KS lesions that also express high levels of the KSHV latency-associated nuclear antigen (LANA) compared to early-stage KS lesions (Qin et al., 2010b). In the same study, we found that KSHV encodes multiple microRNAs that directly target BACH-1, a negative transcriptional regulator of xCT that binds to the *cis*-acting “Antioxidant Response Element” (ARE) within the *xCT* promoter region, thereby upregulating the receptor, which in turn facilitates viral dissemination and enhanced survival of virus-infected cells in the host microenvironment (Qin et al., 2010b). Our findings of a role for KSHV in regulation of its own receptor were the first to demonstrate a direct link between xCT receptor usage and KSHV pathogenesis, and have inspired ongoing work aimed at defining post-entry interactions of KSHV with the human host that can be explored as viable therapeutic targets for clinical management of KS and other virus-associated cancers.

By virtue of its role in maintaining the intracellular redox balance, xCT also protects KSHV-infected cells from death induced by RNS and other insults. Indeed, RNAi silencing of xCT expression impairs the resistance of KSHV-infected mouse macrophage RAW cells to death induced by the nitric oxide (NO) donor, S-nitroso-N-acetylpenicillamine (SNAP; Qin et al., 2010b). We and others also recently reported that overexpression of xCT induces upregulation of 14-3-3β (a downstream regulatory protein from KSHV-infected cells and KS lesions), resulting in intracellular signal transduction via MAPK and increased cytokine release, cell growth, and invasiveness (Zeng et al., 2010; Qin et al., 2013). By enhancing biosynthesis of intracellular GSH, xCT also protects cancer cells from drug-induced oxidative stress by mediating detoxification and extrusion of chemotherapeutic drugs via its biophysical interactions with multidrug resistance proteins (Haimeur et al., 2002; Okuno et al., 2003; Filipits et al., 2005; Yang et al., 2006). For example, GSH induces a conformational change in the multidrug resistance-associated protein-1 (MRP1), which impairs its interaction with one of the most commonly used chemotherapeutic drugs, doxorubicin, and consequently reduces its drug-efflux function (Manciu et al., 2003).

Interestingly, the xCT/CD98 cystine transporter tightly associates with a multi-molecular “supercomplex” on the cell-surface that also includes Emmprin (CD147), LYVE-1 (a hyaluronan receptor), and BCRP (a drug-efflux pump protein responsible for multidrug resistance of KSHV-infected PEL cells; Qin et al., 2011). Within this complex, Emmprin has been reported to confer resistance to some chemotherapeutic drugs (Okuno et al., 2003; Yang et al., 2007; Zou et al., 2007). Indeed, expression of xCT in a panel of cancer cell lines has been associated with potency of 1,400 candidate anticancer drugs, including cisplatin (Huang et al., 2005). Since KSHV induces expression of both xCT and Emmprin in a variety of infected cell lines including PEL cells (Qin et al., 2010a,b; Dai et al., 2012, 2014a), it is probable that stabilization of the xCT/CD98/CD147 supercomplex plays a critical role not only in intracellular energy metabolism (Xu and Hemler, 2005) but also in potentiating the efflux functions of multidrug transporters, which could enhance survival of KSHV-associated cancers including PEL.

### xCT AS A THERAPEUTIC TARGET FOR KSHV-ASSOCIATED LYMPHOMA

As mentioned above, KSHV is a principal causative agent of PEL, which comprises transformed B cells harboring viral episomes. PEL is a rapidly progressing malignancy that arises preferentially within the pleural or peritoneal cavities of patients infected with HIV (Cesarman et al., 1995), with a median survival time of approximately 6 months even under conventional chemotherapy (Chen et al., 2007). xCT is highly expressed in a variety of KSHV-infected PEL cell-lines, and targeting xCT by either RNAi or selective inhibitors induces significant cell apoptosis potentially through regulation of host and viral factors including: (i) reducing intracellular GSH, (ii) increasing ROS, (iii) repressing cell-proliferation-related signaling, and (iv) inducing viral lytic gene expression (Dai et al., 2014a). We also demonstrated that an xCT selective inhibitor, Sulfasalazine (SASP), which has been approved by FDA for treatment of some inflammatory diseases (Gout et al., 2001), can effectively prevent PEL tumor progression in an immune-deficient xenograft mouse model (Dai et al., 2014a,c), supporting the potential benefit of targeting xCT as a strategy for attenuating progression to overt lymphomagenesis. With this as an important goal, additional studies are warranted in order to identify other cell-proliferation/growth-related factors that could be impacted by selective targeting of xCT (including those involved in cell-cycle and autophagy) not only within PEL cells but also in Burkitt’s lymphoma (BL)-derived cell lines where xCT is also highly expressed (Dai et al., 2014a).

## xCT AND HIV PATHOGENESIS

It has been reported that the HIV-encoded transactivator protein Tat can upregulate xCT expression within human primary microglia, retinal pigment epithelium and in retina from Tat-transgenic mice (Bridges et al., 2004; Gupta et al., 2010; Pang et al., 2013), although the underlying mechanisms remain largely unknown. Increased expression of xCT within ocular tissue results in increased release of excitotoxic glutamate in exchange for extracellular cystine, which contributes to the excitotoxicity associated with non-infectious AIDS retinopathy (Bridges et al., 2004; Gupta et al., 2010). In this respect, the direct link between the amino acid transport activity of xCT and retinopathy among HIV-infected individuals suggests that targeting xCT may represent a promising new strategy for ameliorating this disease in high-risk groups (Bridges et al., 2004; Butler and Thorne, 2012).

## xCT AND BACTERIAL INFECTION

The xCT-anchored x_c_^–^ cystine/glutamate transporter plays an important role not only in viral pathogenesis but also during bacterial infection. Thus, xCT mRNA and the cystine/glutamate transporter activity are dramatically induced in mouse peritoneal macrophages *in vitro* by bacterial lipopolysaccharide (LPS) even at very low concentrations, similar to that observed in the plasma of patients with sepsis (Sato et al., 1995). The same group further confirmed that ambient oxygen tension and/or oxidative stress are required for LPS-induced xCT expression and cystine/glutamate transport in mouse peritoneal macrophages *in vitro* (Sato et al., 2001). In an experimental endotoxemia mouse model, xCT mRNA was constitutively expressed in the brain, thymus, and spleen, and xCT mRNA was strongly upregulated in thymus and spleen following administration of a sublethal dose of LPS, indicating that xCT plays an important role during the host inflammatory response to bacterial infection *in vivo* (Taguchi et al., 2007). Interestingly, LPS induction of xCT expression is not mediated by the ARE in the promoter region as described previously (Sasaki et al., 2002); as such, the underlying mechanism for LPS-induced xCT expression requires further investigation.

## CONCLUDING REMARKS

In contrast to extensive studies of the role of xCT as an amino-acid transporter involved in the survival of normal and cancer cells, there are very limited data describing its role in microbial infection and/or the pathogenesis of associated diseases. According to current data, at least 16.1% of cancers worldwide are linked to infectious diseases, and in Sub-Saharan Africa, this rate is almost 32.7% (de Martel et al., 2012). Nonetheless, we note that although the preponderance of infectious cancer is associated with viruses, *Helicobacter pylori*, Mycobacterium, and some parasites have also been associated with a direct or potentiating role in development of some cancers as well (Fried et al., 2011; Pallis and Syrigos, 2013; Mesri et al., 2014; Park et al., 2014). In many cases, studies of the molecular mechanisms by which these infections contribute to the defining markers of cancer initiation and progression have yielded important insights into strategies for control and prevention of infectious disease. More specifically, recent findings from our group and others about the role of xCT in the pathogenesis of KSHV (a model oncogenic virus) have revealed the multi-functional role of xCT not only during primary infection but also in post-entry events that promote infected cell survival and tumorigenesis. Moreover, additional data demonstrating the biologic function of xCT in HIV and bacterial infection further illustrate the broad regulatory role of this protein in various infectious diseases. Therefore, to better understand the breadth of the xCT functional “network”, it will be essential to elucidate: (i) the factors that regulate its expression in various cell types, (ii) the cognate proteins that interact with xCT on cell membrane in response to engagement by exogenous pathogens, (iii) the downstream genes that are controlled by signals initiated by these interactions at the cell surface, and (iv) whether xCT expression in tumors correlates with disease progression and resistance to chemotherapy in cancer-bearing individuals. New information derived from these studies will guide approaches for clinical management of xCT-associated abnormalities, including the development of more specific pharmacological methods for inhibiting or inducing system x_c_^–^ activity as a means for modulating progression of associated disease. With respect to PEL, cell-culture systems such as Nb2-SFJCD1 lymphoma cells (Gout et al., 2001) could be used as platforms for screening small molecule lead-compound libraries for novel xCT inhibitors. Along these lines, it is worth noting that some xCT inhibitors such as SASP have already been approved by FDA for treatment of certain infectious diseases, and its anti-cancer effects are being evaluated in clinical trials (Gout et al., 2001; Robe et al., 2006; Sontheimer and Bridges, 2012).

### Conflict of Interest Statement

The authors declare that the research was conducted in the absence of any commercial or financial relationships that could be construed as a potential conflict of interest.
